# Multicenter Experience with Boceprevir or Telaprevir to Treat Hepatitis C Recurrence after Liver Transplantation: When Present Becomes Past, What Lessons for Future?

**DOI:** 10.1371/journal.pone.0138091

**Published:** 2015-09-22

**Authors:** Audrey Coilly, Jérôme Dumortier, Danielle Botta-Fridlund, Marianne Latournerie, Vincent Leroy, Georges-Philippe Pageaux, Hélène Agostini, Emiliano Giostra, Christophe Moreno, Bruno Roche, Teresa Maria Antonini, Olivier Guillaud, Pascal Lebray, Sylvie Radenne, Anne-Catherine Saouli, Yvon Calmus, Laurent Alric, Maryline Debette-Gratien, Victor De Ledinghen, François Durand, Christophe Duvoux, Didier Samuel, Jean-Charles Duclos-Vallée

**Affiliations:** 1 AP-HP Hôpital Paul-Brousse, Centre Hépato-Biliaire, Villejuif, F-94800, France, Université Paris-Sud, UMR-S 785, Villejuif, F-94800, France, Inserm, Unité 785, Villejuif, F-94800, France, Hepatinov, Villejuif, F-94800, France; 2 Department of Digestive Diseases, Hospices Civils de Lyon, Edouard Herriot Hospital, Lyon, France; 3 Assistance Publique—Hôpitaux de Marseille, Centre Hospitalo-Universitaire Conception, Service d'Hépato-Gastro-Entérologie, Marseille, France; 4 CHU Rennes, Liver Disease Unit, Rennes, France; 5 Service d’hépato-gastro-entérologie, hôpital A.-Michallon, 38700, La Tronche, France; 6 Fédération médico-chirurgicale des maladies de l’appareil digestif, hôpital Saint-Eloi, 34295, Montpellier, France; 7 AP-HP, Hôpital Bicêtre, Unité de recherche clinique Paris-Sud, Kremlin-Bicêtre, France; 8 Department of Gastroenterology and Hepatology, Geneva University Hospital, Rue Gabrielle-Perret-Gentil 4, 1211, Geneva, 14, Switzerland; 9 Liver unit, Department of Gastroenterology, Hepatopancreatology and Digestive oncology, CUB Hôpital Erasme, Université Libre de Bruxelles, Brussels, Belgium; 10 Assistance Publique Hôpitaux de Paris (AP-HP), Groupe Hospitalier Pitié-Salpêtrière, Paris, France; 11 Service d’hépatologie, HCL, hôpital de la Croix-Rousse, 69205, Lyon, France; 12 Hepato-Bilio-Pancreatic Surgery and Liver Transplantation Center, Université de Strasbourg, Strasbourg, France; 13 Department of Hepatobiliary and Liver Transplantation Surgery, Hopital Saint Antoine, Assistance publique-Hopitaux de Paris, 184 rue du Faubourg Saint-Antoine, 75012, Paris Cedex, France; 14 Internal medecine-Digestive department UMR 152 IRD Toulouse 3 University, Toulouse, France; 15 Service d'Hépato-gastroentérologie, CHU de Limoges, 2 avenue Martin-Luther-King, 87042, Limoges, France, Inserm UMR 1092, Faculté de médecine de Limoges, Université de Limoges, Limoges, France; 16 University of Bordeaux, Bordeaux, France; 17 Service d’hépatologie, hôpital Beaujon, AP–HP, 92118, Clichy, France; 18 Service d’hépatologie, hôpital Henri-Mondor, AP–HP, 94000, Créteil, France; Kaohsiung Medical University Hospital, Kaohsiung Medical University, TAIWAN

## Abstract

**Background and aims:**

First generation protease inhibitors (PI) with peg-interferon (PEG-IFN) and ribavirin (RBV) have been the only therapy available for hepatitis C virus (HCV) genotype 1 infection in most countries for 3 years. We have investigated the efficacy and tolerance of this triple therapy in transplanted patients experiencing a recurrence of HCV infection on the liver graft.

**Patients:**

This cohort study enrolled 81 liver transplant patients (Male: 76%, mean age: 55.8±9.7 years) with severe HCV recurrence (F3 or F4: n = 34 (42%), treatment experienced: n = 44 (54%)), treated with boceprevir (n = 36; 44%) or telaprevir (n = 45; 56%). We assessed the percentages of patients with sustained virological responses 24 weeks after therapy (SVR24), and safety.

**Results:**

The SVR24 rate was 47% (telaprevir: 42%; boceprevir: 53%, *P* = ns). At baseline, a normal bilirubin level (*p* = 0.0145) and albumin level >35g/L (*p* = 0.0372) and an initial RBV dosage of ≥800 mg/day (*p* = 0.0033) predicted SVR24. During treatment, achieving an early virological response after 12 weeks was the strongest independent factor to predict SVR24 (*p*<0.0001). A premature discontinuation of anti-HCV therapy due to a serious adverse event (SAE) was observed in 22 patients (27%). Hematological toxicity, infections and deaths were observed in 95%, 28% and 7% of patients, respectively. A history of post-LT antiviral therapy and thrombocytopenia (<50G/L) during treatment were both independent predictors of the occurrence of infections or SAE (*p* = 0.0169 and *p* = 0.011).

**Conclusions:**

The use of first generation PI after liver transplantation enabled an SVR24 rate of 47% in genotype 1 patients, but induced a high rate of SAE. The identification of predictive factors for a response to treatment, and the occurrence of SAE, have enabled us to establish limits for the use of this anti-HCV therapy in the transplant setting.

## Introduction

End-stage liver disease and hepatocellular carcinoma due to hepatitis C virus (HCV) infection constitute one of the main indications for liver transplantation (LT) worldwide [1]. In France, it accounts for more than 10% of LT per year according to the National Biomedical Agency (www.agence-biomedecine.fr/). The European Liver Transplant Register reports that overall and graft survival rates range from 61% to 65%, and 57% to 60% at five-years post-LT, respectively, depending on the indication with or without hepatocellular carcinoma (HCC) [2]. HCV recurrence on the liver graft is responsible for these poor results as it is the leading cause of graft loss and post-LT mortality [3]. HCV recurrence is constant when patients are transplanted with a positive HCV viral load (VL), and affects 20% of patients when they are transplanted with a negative VL but did not achieve a sustained virological response (SVR) pre-LT [4]. Dual therapy based on peg-interferon (PEG-IFN) and ribavirin (RBV) has been the standard of care for HCV recurrence during the past ten years. Patients who achieved an SVR had a better outcome [5], but this only concerned 30% of patients. This was essentially related to poor tolerance, which led to a 30% premature discontinuation rate [6].

In 2011, introduction of the first generation protease inhibitors (PI), boceprevir (BOC) and telaprevir (TVR), marked a new era of direct antiviral agent (DAA)-based regimens to treat HCV, improving SVR rates in non-transplant genotype 1 (G1) patients [7]. Their use after LT was initially decried because of potent drug-drug interactions between PI and immunosuppressive drugs (IS) [8,9]. Early reports demonstrated that these interactions could be managed easily through the close monitoring of trough blood concentrations (C0) of IS such as calcineurin inhibitors (CNI) [10]. Several studies were carried out worldwide to assess the safety and efficacy of triple therapy based on first generation PI after LT [11,12,13,14,15]. They described some concerns with respect to tolerance (infections, cytopenia) but improvements in virological responses. None of these reports offered tools to guide treatment initiation or to limit this use.

The main objective of the present study was to describe factors for efficacy as a function of the SVR24 and safety of triple therapy based on first generation PI to treat HCV recurrence after LT in a large cohort of patients.

## Methods

### Study design

This prospective study was performed in a real-life cohort approved by the Transplantation Prospective Group of the Association Française pour l’Etude du Foie (AFEF, French Association for the Study of the Liver) in October 2011. This was an observational study. The physicians chose to treat patients for HCV recurrence in accordance with current guidelines. Patients were enrolled in 17 transplant centers in France, Belgium and Switzerland. They were included when at least one dose of anti-HCV therapy was administered. Data were collected prospectively between March 2011 and January 2014.

### Patients

Transplanted patients who experienced a G1 HCV recurrence and were treated with a triple therapy based on PEG-IFN/RBV and BOC or TVR between March 2011 and January 2014 were thus studied. The genotypes were determined using phylogenetic analysis of the NS5B region [16]. All patients treated during this period were enrolled and gave their written consent to being included in the study. The indication for antiviral therapy was based on biopsy-proven chronic hepatitis defined using the METAVIR score. All the patients included were suffering from a biopsy-proven HCV recurrence with fibrosis stage ≥F1 or from cholestatic hepatitis (CH), defined according to the following criteria [17]; *i*.*e*. the presence of extensive, dense portal fibrosis with immature fibrous bands extending into the sinusoidal spaces, ductular proliferation, cholestasis and moderate mononuclear inflammation. The exclusion criteria were HIV co-infection and contraindications to the use of one of the antiviral drugs, including uncontrolled biopsy-proven acute rejection (BPAR). Cirrhotic patients were enrolled when compensated.

Data were collected on the testing of recipient DNA for interleukin (IL) 28B polymorphism rs12979860C/T using the ABI TaqMan allelic discrimination kit and the ABI7900HT Sequence Detection System (Applied Biosystems, Carlsbad, CA).

Before the initiation of PI, a minimal calcineurin inhibitor (CNI) trough concentration (C0) was targeted and all patients were at a steady state regarding the IS. Whole blood concentrations were assayed using a chemiluminescent microparticulate immunoassay on an Architect autoanalyzer for CNI, and liquid chromatography coupled to tandem mass spectrometry for everolimus. The laboratory was a participant in an international external quality control scheme (Analytical Services International Ltd, London). Most patients were hospitalized the day before PI initiation in order to enable their close clinical monitoring. Daily controls of CNI C0 were constantly measured until a therapeutic target range was obtained. The IS dosing regimens were adjusted to reach a therapeutic range that differed as a function of the time post-LT and the previous BPAR episode. The C0 ranged from 50 to 150 ng per milliliter (mL) for cyclosporine, from 5 to 10 ng/mL for tacrolimus and from 3 to 8 ng/mL for everolimus. During triple therapy, C0 were monitored at every visit. At the end of PI therapy, C0 were once again monitored on a daily basis until a steady state was achieved.

All patients received PEG-IFN/RBV. Both PEG-IFNα2a (Pegasys®; Roche) and PEG-IFNα2b (Viraferon-peg®; Schering-Plough) were used in line with the decision of the senior referent physician. PEG-IFNα2a and RBV (Copegus®; Roche, Rebetol®; Schering-Plough) doses were individualized based on the patient's weight and adjusted to renal function parameters and the blood count. Doses could be escalated to maximally tolerated levels, or reduced depending on overall tolerance. Each investigator decided which PI should be used in light of the patient's need and their own experience. BOC became available before TVR in France thanks to an early access program which started in early 2011. BOC (800 mg tid) was always initiated after a 4-week (W) lead-in phase, while PEG-IFN/RBV. TVR (750 mg tid) was usually initiated with PEG-IFN/RBV at the same time but a lead-in phase was also ensured before the introduction of TVR when it was necessary to assess hematological and renal tolerance. The intended duration of therapy was 48 W. The stopping rule applied was failure to achieve a reduction in the HCV viral load (VL) to less than 100 IU/mL at W12 in the BOC group, and to less than 1000 IU/mL in the TVR group; in the event of such a non-response, all treatment was discontinued and the patient advanced to follow-up. Erythropoietin (EPO) (Neorecormon®; Roche) was administered to support the red blood cell count when hemoglobin levels dropped below 10g/dL, or decreased by >1g/dL/W, or when a transfusion had been necessary during prior antiviral therapy. In some cases, an RBV dose reduction was required, but this depended on the practices of individual physicians. Granulocyte colony stimulating factor (G-CSF) (Neupogen®, Amgen Europe BV) was usually administered to support the neutrophil count when it fell below 750 cells/mm3 despite a reduction in the PEG-IFN dose.

Our experience concerning the first 37 patients was reported elsewhere regarding the preliminary efficacy and safety results [17].

### Assessments of efficacy

Viral loads were monitored in plasma using the Abbott Real Time HCV assay (Abbott Molecular, USA; lower limit of detection: 12 IU/mL) at baseline, at weeks 4, 8, 12, 24 and 48 during therapy and at weeks 4, 12 and 24 after therapy. In the event of virological breakthrough, and when sera were available, the entire NS3 region was analyzed by sequencing to detect any PI resistance mutations.

### Efficacy end points

The primary efficacy end point was a sustained virological response (HCV RNA <12 IU/mL) at 12 W (SVR12) and at 24 W (SVR24) after treatment discontinuation. Secondary efficacy end points included: (i) a rapid virological response (RVR) defined as an undetectable VL at W4 of triple therapy, (ii) a complete early virological response (EVR) defined as an undetectable VL at W12, (iii) an extended rapid virological response (eRVR) defined as an undetectable VL at W4 and W12 of triple therapy, and (iv) an end of treatment therapeutic response (EOT) defined as a negative VL at the discontinuation of therapy. All these virological responses were obtained in the intention-to-treat population. A viral breakthrough (VB) was defined as achieving an undetectable VL but then either a subsequent occurrence of a detectable VL higher than 2-log_10_ IU/mL, or a 1-log_10_ IU/mL increase in VL over time.

### Safety assessments

Adverse events were assessed at each consultation visit: at baseline, at weeks 4, 8, 12, 24 and 48 during therapy and at weeks 4, 12 and 24 after therapy. However, investigators were able to schedule supplementary visits when the condition of their patients required close follow-up; for example, if any adverse events occurred. For the purposes of this paper, serious adverse events (SAE) were defined as all unexpected events occurring during treatment whatever their cause, and required treatment discontinuation. Severe cytopenia was defined as cytopenia requiring treatment discontinuation.

Data on the type of SAE and on clinical and biological parameters were collected during the period of antiviral therapy and for 6 months after administration of the last dose. The estimated glomerular filtration rate (eGFR) was assessed using the Cockcroft-Gault formula [18].

The investigators managed all adverse events according to AFEF guidelines [19].

### Statistical Analysis

Analyses were performed in the modified intention-to-treat population, which included all patients who had received at least one dose of anti-HCV therapy. SAS software, Version 9.3 (SAS Institute Inc., Cary, NC, USA) was used for these analyses. All statistical tests and 95% confidence intervals were two-sided, with a significance level of 0.05. When data were supplied as mean values, ranges were provided [minimum-maximum] and when data were supplied as median values, interquartile ranges were provided [IQR1-IQR3]. Differences in baseline characteristics between the two treated groups were evaluated using the chi-square test for categorical data and one-way analysis of variance for continuous data. Comparisons of efficacy, adverse events and laboratory abnormalities were performed using Fisher’s exact test. The relationships between specified baseline characteristics and the sustained response rates at post-treatment weeks 12 and 24, and the occurrence of adverse events, were analyzed by means of stepwise logistic regression to determine independent predictors for a sustained virological response at W12 and W24 and the occurrence of adverse events. Covariates with a p-value<0.2 in univariate analysis were included in the multivariate model.

## Results

### Patients

Eighty-one patients were enrolled in 17 centers. The principal baseline demographic and disease characteristics of the studied population are shown in Table 1. Of the 81 patients, 61 (76%) were men and their mean age was 55.8±9.7 years [33.0–74.0]. In terms of the severity of HCV recurrence, 19 (24%) were cirrhotic patients, and nine (11%) experienced a CH. Forty-four (54%) had previously been treated with PEG-IFN/RBV after LT. Their prior therapeutic responses had been a relapse or virological breakthrough in 16 cases (20%), a partial response in ten cases (12%) and a null response in 18 cases (22%). Thirty-seven (46%) patients were naive of anti-HCV therapy after LT. Thirty-six (44%) and 45 (56%) patients have received BOC and TVR, respectively. Fifty-five patients (68%) completed a 4W lead-in phase, including 19 patients in the TVR group. The median time elapsing between LT and triple therapy was 36 months [24–84]. The mean duration of antiviral therapy was 42.7±37.7 W [1–49], and was similar in both groups (*P* = 0.87). As for immunosuppressive regimens, there were no differences in the choice of CNI and the number of immunosuppressive drugs between the BOC and TVR groups (Table 2). At baseline, 76 patients (94%) received a CNI. Most patients received at least two or three immunosuppressive agents.

**Table 1:** Baseline characteristics of the study population. Continuous variables are expressed as mean ± standard deviation.

	Overall population (n = 81)	Boceprevir (n = 36)	Telaprevir (n = 45)	*P*
Age (years)	55.8±9.7	54.8±11.0	56.1±8.9	ns
Gender (M)–n (%)	68 (81%)	28 (78%)	40 (89%)	ns
Body Mass Index (Kg/m^2^)	24.8±3.9	24.6±3.8	24.3±3.5	ns
Indication for LT—n (%)				
End-stage liver disease	33 (41%)	18 (50%)	15 (33%)	ns
HCC	41 (51%)	15 (42%)	26 (58%)	Ns
HCV ReLT	7 (9%)	3 (8%)	4 (9%)	ns
Combined liver/kidney transplantation	3 (4%)	2 (6%)	1 (2%)	ns
HBV/HCV co-infection—n (%)	1 (1%)	0 (0%)	1 (2%)	ns
Treatment history—n. (%)				
Treatment-naive patients	37 (46%)	16 (44%)	21 (47%)	ns
Null-responders	18 (22%)	7 (20%)	11 (25%)	ns
Partial responders	10 (12%)	5 (14%)	5 (11%)	ns
Relapsers	11 (14%)	5 (14%)	6 (13%)	ns
Virological breakthrough	5 (6%)	3 (8%)	2 (4%)	ns
Interval between LT and antiviral therapy (years)	4.8±5.0	4.8±5	5.1±4.3	ns
HCV Genotype—n (%)				
1a	25 (30%)	13 (36%)	12 (26%)	ns
1b	55 (70%)	23 (64%)	32 (72%)	ns
Recipient IL-28b rs12979860 Genotype—n (%)				
CC	7 (9%)	6 (17%)	1 (2%)	ns
CT	23 (28%)	12 (34%)	11 (25%)	ns
TT	13 (16%)	9 (25%)	7 (15%)	ns
Undetermined	35 (43%)	9 (25%)	26 (58%)	ns
Fibrosis stage (Metavir)–n (%)				
≤F2	47 (58%)	23 (64%)	24 (53%)	ns
≥F3	34 (42%)	23 (64%); 13 (36%)	24 (53%); 21 (47%)	ns; ns
F4	19 (23%)	9 (25%)	10 (22%)	Ns
Cholestatic hepatitis—n (%)	9 (11%)	2 (6%)	7 (16%)	Ns
MELD score	11.7±13.0	18.1±18.8	9.4±4.28	0.007
Total bilirubin (μmol/L)	32.8±51.2	35.9±61.4	31.3±51.0	Ns
ALT (IU/L)	140.0±158.5	172.6±179.6	84.4±83.0	0.006
Albuminemia (g/L)	36.3±5.9	36.7±5.7	35.7±6.5	Ns
Creatinine clearance (mL/min)	104.2±32.3	103.3±31.7	105.6±28.3	Ns
Hemoglobin (g/dL)	12.5±2.3	13.8±1.4	13.7±1.8	Ns
Neutrophil count (G/L)	3.6±5.2	4.1±6.4	3.1±1.7	Ns
Platelet count (G/L)	129.5±60.2	138.1±75.8	149.1±51.8	Ns
HCV viral load (log_10_ IU/mL)	6.6±0.7	6.7±0.7	6.6±0.7	Ns
PEG-IFN alpha-2a –n (%)	63 (78%)	19 (53%)	44 (98%)	<0.0001
Ribavirin dosage (mg/day)	792.0±266.0	793.9±271.5	790.5±264.9	Ns
Lead-in phase—n (%)	55 (68%)	36 (100%)	19 (45%)	<0.0001

Abbreviations: HBV: hepatitis B virus; HCC: hepatocellular carcinoma; HCV: hepatitis C virus; M: male; n: number; PEG-IFN: pegylated interferon

**Table 2:** Description of immunosuppressive therapy management.

	Boceprevir (n = 36)	Telaprevir (n = 45)	*P*
**Number of IS drugs—n (%)**			
1	16 (44%)	24 (53%)	ns
2	15 (42%)	19 (42%)	Ns
3	5 (14%)	2 (4%)	ns
**CNI, n (%)**			
Cyclosporine	19 (53%)	24 (53%)	ns
Tacrolimus	14 (39%)	19 (42%)	ns
**mTOR inhibitors, n (%)**			
Everolimus	2 (6%)	1 (2%)	ns
Sirolimus	3 (8%)	1 (2%)	ns
**Prednisone, n (%)**	12 (33%)	7 (16%)	ns
Dosage (mg/day), mean±sd	8.0±4.9	4.4±2.4	ns
**MMF—n (%)**	17 (47%)	20 (44%)	ns
Dosage (mg/day), mean±sd	1411.8±754.9	847.2±496.9	0.01
**CNI fold reduction at PI initiation, mean±sd**			
Cyclosporine	1.9±1.0	2.5±1.3	NA
Tacrolimus	4.8±3.1	29.4±19.6	NA
**CNI fold increase at PI discontinuation, mean±sd**			
Cyclosporine	1.2±0.8	2.2±1.4	NA
Tacrolimus	4.9±2.4	26.2±24.3	NA

Abbreviations: CNI: calcineurin inhibitors; IS: immunosuppression; n: number; sd: standard deviation; MMF: mycophenolate mofetil; PI: protease inhibitors

### Efficacy

#### Biological improvements

Forty-five patients (56%) presented with elevated bilirubin levels prior to treatment (mean: 33.4±55.6 μmol/L [7–372]). Among these, 25 (55%) reduced, 15 (33%) stabilized and five (11%) increased their bilirubin levels at the end of therapy, respectively. When normal at baseline, no patient experienced an increase of bilirubin level up to 50 μmol/L during treatment and achieved normalization when treatment was discontinued.

At baseline, the median MELD score was 9.5 [6.0–12.6]. At the end of therapy, this MELD score was 7.2 [6.0–9.5] (*p* = ns). The reduction in the MELD score was particularly significant in patients with CH and in those with an F4 METAVIR score: the median MELD scores fell from 12.1 [10.0–13.4] to 8.2 [6.0–9.2] and from 12.0 [9.5–13.9] to 9.1 [7.2–11.7], respectively (*p* = 0.021 and *p* = 0.047).

#### Virological responses (Fig 1)

**Fig 1:**
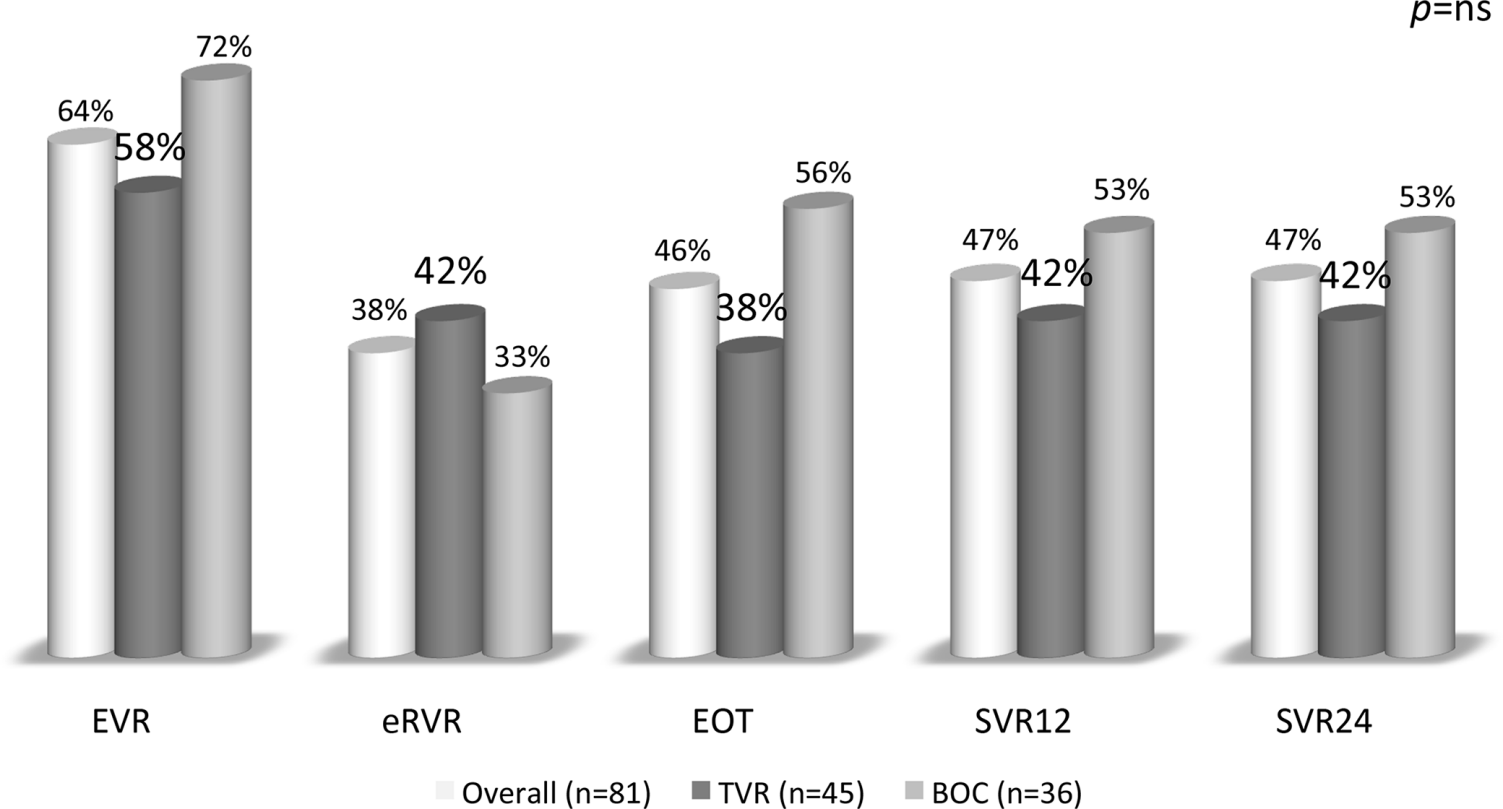
Virological responses during triple therapy after liver transplantation. An early virological response (EVR) was observed when the HCV viral load was undetectable at week 12. An extended virological response meant negative HCV RNA at week 4 and week 12. An EOT (end of treatment response) was achieved when HCV RNA was undetectable at 48 weeks. SVR12 and SVR 24 were defined as undetectable HCV RNA at 12 and 24 weeks after the discontinuation of antiviral therapy, respectively. Five patients discontinued triple therapy (three with TVR and two with BOC) before week 48 with undetectable HCV RNA and still achieved an SVR12 and SVR24.

An EVR and eRVR were achieved in 52 (64%) and 31 (38%) patients, respectively. Thirty-eight patients (47%) achieved a SVR12 and SVR24 in this cohort, 19 (53%) with BOC and 19 (42%) with TVR (p = 0.344). Stepwise logistic regression showed that achieving an EVR was the most robust independent factor for SVR12 and SVR24 (OR = 49.4, 95%CI [5.7–421.3], p<0.00001). Among the 52 patients with an EVR, 38 (73%) achieved a SVR24. A lead-in phase did not impact the SVR24 (p = 0.821).

Baseline characteristics such as a bilirubin level <17μmol/L (OR = 5.5, 95%CI [1.4–21.4], *p* = 0.015), an albumin level >35g/L (OR = 4.6, 95%CI [1.1–19.3], *P* = 0.037) and an initial dosage of RBV dosage of ≥800mg/day (OR = 9.8, 95%CI [2.1–44.6], *P* = 0.003) had a significant and positive impact on both the SVR12 and SVR24 (Table 3). If no premature discontinuation of therapy due to SAE was necessary, this also had an impact on SVR rates (OR = 6.3, 95%CI [1.3–31.1], p = 0.022).

**Table 3:** Univariate and multivariate analysis of factors related to SVR24. Covariates with a p-value<0.2 under univariate analysis were included in the multivariate model.

		Univariate analysis	Multivariate analysis			
	%SVR24	*P*	*P*	Odds ratio	95% CI	
**Before treatment**						
Recipient IL28B status (CC/CT/TT)	86/57/38	0.098				
BMI (Kg/m2) >25	50	0.099				
Fibrosis stage ≤F2 vs >F2	53 vs 30	0.060				
Null response post-LT	47	0.190				
**Bilirubin level <17μmol/L**	28	**0.013**	**0.015**	**5.484**	**1.402**	**21.453**
**Albumin level >35g/L**	54	**0.026**	**0.037**	**4.604**	**1.094**	**19.366**
MELD score ≤10	54	0.024				
Cyclosporine use	50	0.014				
No steroid use	47	0.040				
Boceprevir use	52	0.087				
**Baseline RBV dose ≥800mg/day**	58	**0.004**	**0.003**	**9.775**	**2.139**	**44.673**
**During treatment**						
**EVR**	70	**<0.00001**	**0.001**	**49.394**	**5.791**	**421.277**
**No premature discontinuation for SAE**	66	**0.04**	**0.022**	**6.360**	**1.300**	**31.115**
Treatment duration	73	0.08				

Abbreviations: BMI: body mass index; EVR: early virological response; RBV: ribavirin; SAE: serious adverse event; SVR24: sustained virological response 24 weeks after treatment discontinuation; vs: versus.

Concerning the immunosuppressive regimen, the use of cyclosporine and the avoidance of steroids were both significantly associated with a SVR24 under univariate analysis (*p* = 0.014 and *p* = 0.040, respectively).

Depending on the response to a previous course of PEG-IFN/RBV therapy after LT, there was a trend towards a lower SVR24 rate among null-responders when compared to relapsers (6/18 (33%) *versus* 6/11 (53%), *P* = 0.190).

It was not possible to detect any influence of genotype 1a (11/25 (44%)) *versus* 1b (26/55 (47%), *P* = 0.814), recipient IL28B polymorphism (*P* = 0.098) or baseline HCV VL (*P* = 0.582) on SVR24.

Depending on the fibrosis stage, a SVR24 was achieved in 26/47 (57%) of ≤F2 patients and in 12/34 (35%) of ≥F3 patients (*P* = 0.06). The SVR24 rate was lower in cirrhotic patients (7/19 (37%)) and among the nine FCH patients, while three patients (33%) achieved an SVR24.

**Fig 2:**
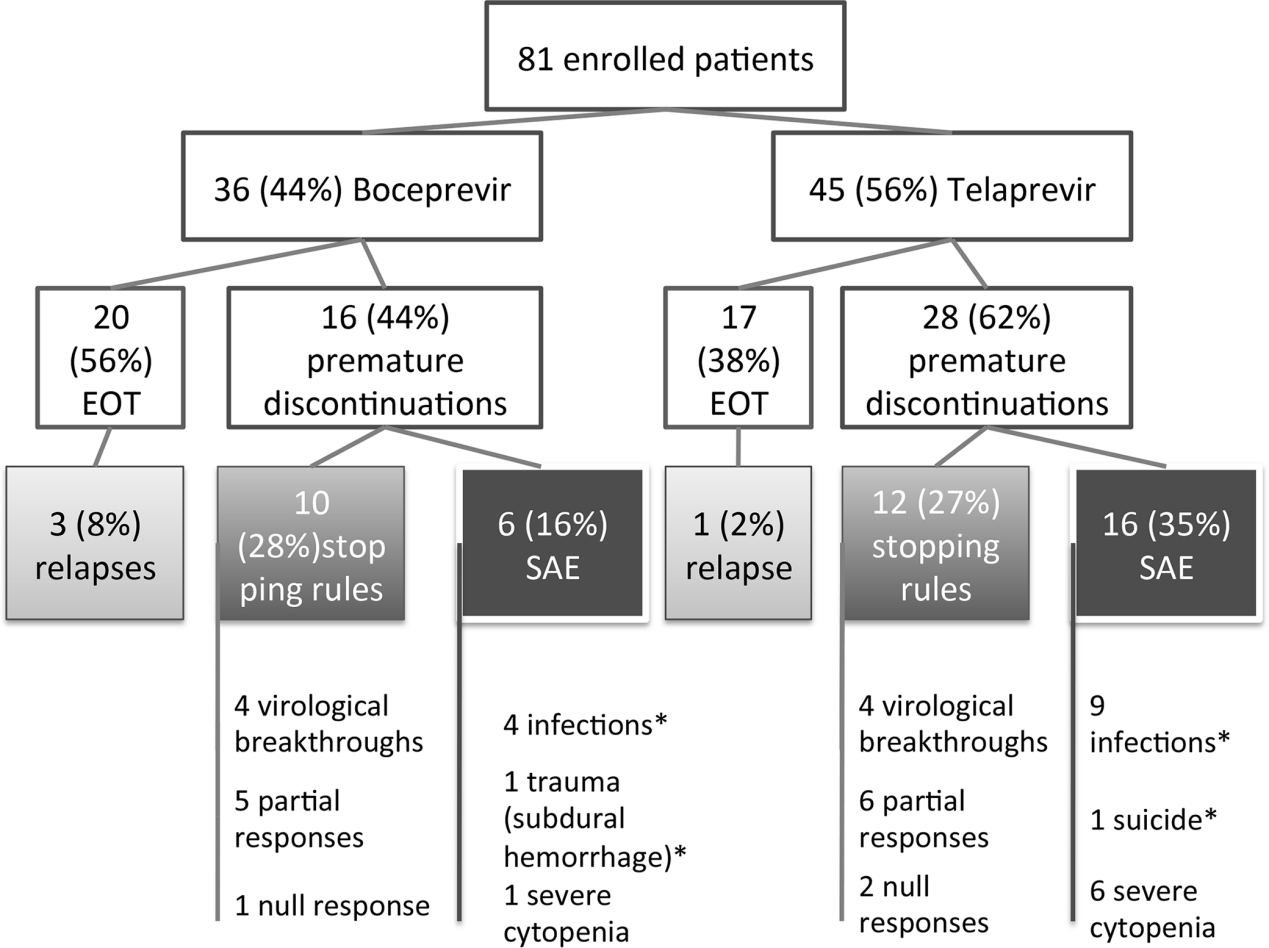
Flowchart describing premature discontinuations and treatment failures. Among the patients who discontinued treatment because of adverse events, five nevertheless achieved an SVR24 (two in the BOC group and three in the TVR group).* indicates the causes of death in this cohort: four deaths from infection (two in each group) and two other causes as shown here.

Premature discontinuation according to the stopping rules occurred in 22 patients (27%) (Fig 2). Among the eight patients who experienced a virological breakthrough, complete NS3 sequencing was performed in six of them and at least one mutation related to PI resistance was detected in three (50%) (T54A, V36M, R155K). Three (8%) and one (2%) patients experienced a relapse after completing 48W of therapy with BOC or TVR, respectively (*P* = 0.11). All relapses occurred during the first 12W following treatment discontinuation.

### Safety

Premature discontinuations due to SAE occurred in 22 patients (27%); six (16%) and 16 (35%) patients in the BOC and TVR groups, respectively (*P* = 0.126) (Fig 2).

A high proportion of hematological events (95%) was observed to lead to treatment discontinuation in seven patients (8%) (Table 4). Twenty-five episodes of infection were reported in 23 patients (28%), affecting 11/36 (31%) and 12/45 (27%) patients receiving BOC and TVR, respectively (*p* = ns) (S1 Table). Thirteen patients (16%) discontinued their triple therapy because of an infection. Four independent factors were associated with the onset of infection (Table 5): patients with CH (OR = 5.9, 95%CI [1.1–33.9], *P* = 0.046), the use of cyclosporine (OR = 4.7, 95%CI [1.2–18.2], *P* = 0.025), a baseline eGFR <60mL/min (OR = 4.7, 95%CI [1.2–17.8], *P* = 0.023) and the occurrence of thrombocytopenia (platelet count <50G/L) during therapy (OR = 5.7, 95%CI [1.6–20.8], *P* = 0.008). Four patients (5%) died during the treatment as the result of an infectious episode (Table 5). One patient died from a subdural hematoma in the context of a trauma and one other patient committed suicide.

**Table 4:** Hematological adverse events and their management during triple therapy after liver transplantation.

	Overall population (n = 81)	Telaprevir group (n = 45)	Boceprevir group (n = 36)	*P*
**Events**				
Hb < 10 g/dL (%)	95%	94%	96%	ns
Hb < 8 g/dL (%)	49%	61%	40%	0.075
NC < 1 G/L (%)	54%	72%	40%	0.004
NC < 0.5 G/L (%)	9%	9%	10%	ns
PC < 50 G/L (%)	38%	53%	27%	0.02
PC < 25 G/L (%)	7%	9%	5%	ns
**Management**				
EPO use (n/%)	76/94%	42/93%	34/94%	ns
Mean delay of between baseline and initiation	3.8±7.4 W	4.2±8.9 W	3.1±4.6 W	ns
RBV reduction (n/%)	56/70%	26/72%	30/67%	ns
Median delay between baseline and the 1st reduction	5 W	8 W	4 W	ns
Red blood cell transfusion (n/%)	32/40%	16/36%	16/44%	ns
Mean delay between baseline and initiation	8.7±8.5 W	7.4±6.1 W	9.8±10.2 W	ns
Mean number of units used during therapy	4.2±2.7	4.7±3.0	3.5±1.9	ns
GCSF use (n/%)	15/18%	6/13%	9/25%	ns
Mean delay of initiation from baseline	5.5±8.7 W	3.2±4.2 W	7.2±11.0 W	ns
Elthrombopag use (n/%)	5/6%	2/4%	3/9%	ns
Mean delay between baseline and initiation	4.2±4.6 W	1.5±2.1 W	6.0±5.2 W	ns
PEG-IFN reduction (n/%)	31/39%	18/50%	13/29%	ns
Mean delay between baseline and reduction	8 W	10.5 W	2 W	ns

Abbreviations: EPO: erythropoietin; Hb: hemoglobin level; NC: neutrophil count; ns: non significant; PC: platelet count; PEG-IFN: pegylated interferon; RBV: ribavirin; W: week

**Table 5:** Predictive factors related to the occurrence of episodes of infection. Covariates with a p-value<0.2 under univariate analysis were included in the multivariate model.

		Univariate analysis	Multivariate analysis			
	% of infection	*P*	*P*	Odds Ratio	CI 95%	
Acute rejection before the introduction of triple therapy	67	0.007				
Previous course of antiviral therapy post-LT	20	0.137				
**Cholestatic hepatitis**	**56**	**0.055**	**0.047**	**5.903**	**1.028**	**33.905**
**Cyclosporine use**	**35**	**0.168**	**0.025**	**4.708**	**1.212**	**18.291**
Number of immunosuppressive drugs	28/24/57	0.196				
Baseline bilirubin level >17μmol/L	38	0.052				
**Baseline creatinine clearance <60mL/min**	**40**	**0.080**	**0.023**	**4.695**	**1.241**	**17.766**
Baseline albumine level < 35g/L	41	0.139				
Anaemia <8g/dL during treatment	40	0.022				
**Thrombocytopenia <50G/L during treatment**	**45**	**0.008**	**0.008**	**5.718**	**1.571**	**20.811**

Abbreviation: LT: liver transplantation

When considering all the SAE and infections that gave rise to a treatment discontinuation, the most robust predictors were the occurrence of thrombocytopenia (platelet count <50G/L) during therapy (OR = 4.4, 95%CI [1.4–13.8], *P* = 0.011) and patients who had failed a previous course of dual therapy after LT (OR = 4.0, 95%CI [1.3–12.4], *P* = 0.017) (Table 6).

**Table 6:** Predictive factors related to occurrence of infections or serious adverse events*. Covariates with a p-value<0.2 under univariate analysis were included in the multivariate model.

		Univariate analysis	Multivariate analysis			
	% of SAE	*P*	*P*	Odds Ratio	CI 95%	
Acute rejection before the introduction of triple therapy	67	0.077				
**Previous course of antiviral therapy post-LT**	**27**	**0.014**	**0.0169**	**3.993**	**1.282**	**12.440**
Cholestatic hepatitis	67	0.077				
Steroids use	53	0.181				
Tacrolimus use	27	0.062				
MMF dosage	46	0.179				
Lead-in phase	33	0.070				
Initial dosage of PEG-IFN	44	0.162				
Baseline MELD score ≥10	52	0.016				
Baseline hemoglobin level >10g/dL	36	0.192				
Baseline bilirubin level >17μmol/L	48	0.087				
Baseline creatinine clearance <60mL/min	33	0.161				
Baseline albumin level <35g/L	52	0.064				
Anaemia <8g/dL during treatment	50	0.056				
**Thrombocytopenia <50G/L during treatment**	**58**	**0.007**	**0.0111**	**4.409**	**1.404**	**13.846**

Abbreviations: LT: liver transplantation; MMF: mycophenolate mofetil; PEG-IFN: pegylated interferon.

* As mentioned in the “Patients and methods” section, a serious adverse event was defined as an unexpected event occurring during treatment and giving rise to treatment discontinuation.

**Fig 3:**
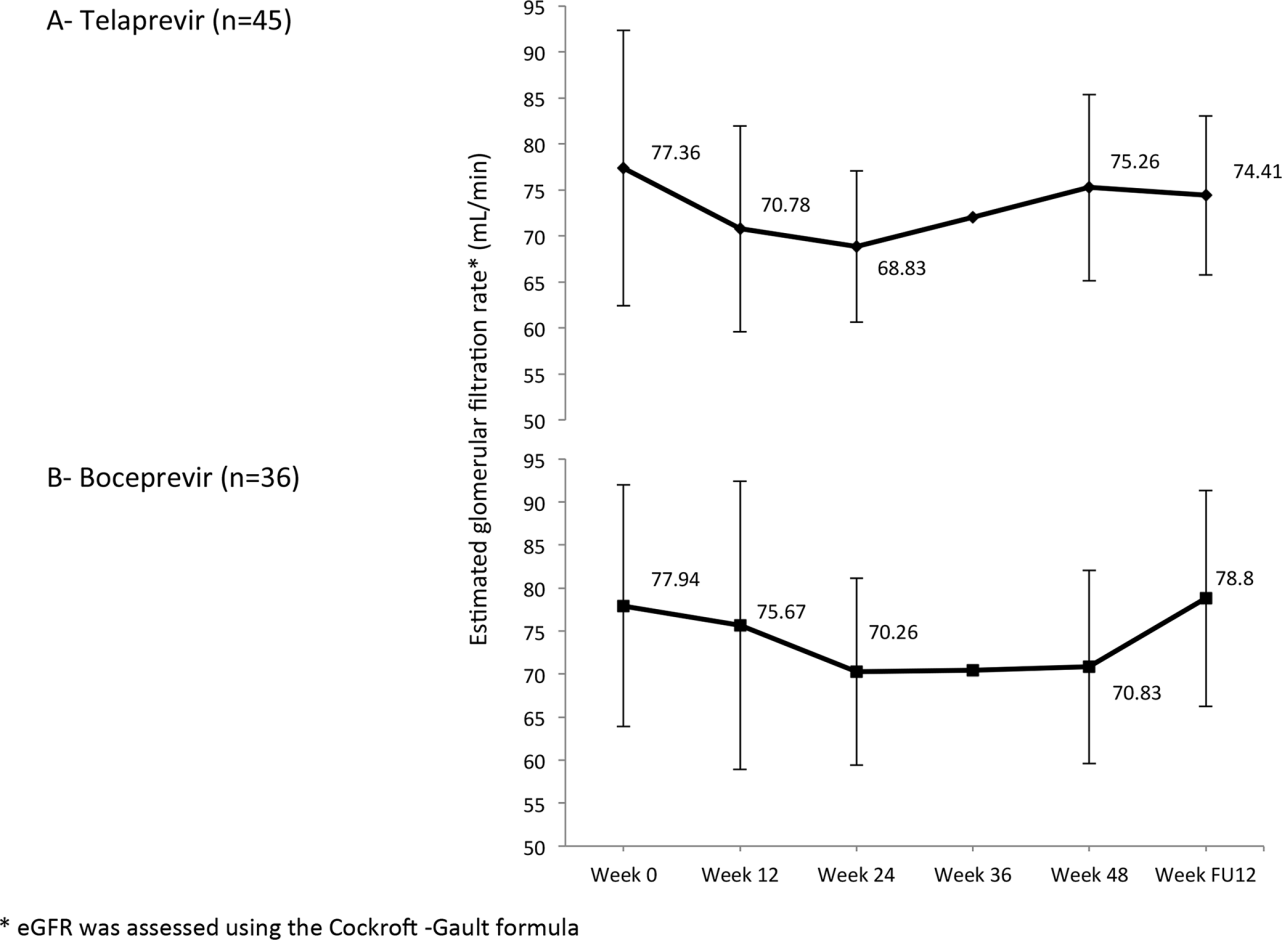
Mean estimated glomerular filtration rates during triple therapy. The median decrease of eGFR was 7.68mL/min with BOC and 8.53mL/min with TVR (*P* = ns). Seven patients were hospitalized because of acute kidney failure, six of whom were in the TVR group. No CNI overdoses were observed and all patients recovered after a saline infusion.

The kinetics of eGFR during treatment is shown in Fig 3. The median decrease of eGFR was 7.68 mL/min with BOC and 8.53 mL/min with TVR (*P* = ns). Seven patients were hospitalized because of acute kidney failure, six of whom were receiving TVR. No CNI overdoses were observed and all patients recovered after a saline infusion.

Five (6%) dermatological events (grade 1) and 2 (3%) cases of decompensated diabetes mellitus were observed. The hospitalization rate during therapy was 44% (n = 36). Ten (12%) acute rejections (median BANFF score 3) were histologically proven in this cohort, six (17%) in the BOC group and four (9%) in the TVR group, respectively (median interval between the introduction of PI and BPAR of 16 W).

## Discussion

This study of a multicenter cohort reports for the first time on the efficacy with SVR24 results and on tolerance of anti-HCV therapies, including first generation PI, in difficult-to-treat patients after LT. This experience has provided us with several key elements that can guide treatment decisions and patient management during this therapy.

In terms of efficacy, these findings have demonstrated that the combination of BOC or TVR with standard PEG-IFN and RBV therapy can add a benefit in terms of SVR24, the rate achieved being 47% when compared with the expected SVR rate of 30% under dual therapy [6]. These results should be considered in the context of this particular study population, which included a high proportion of patients with advanced fibrosis. Indeed, this cohort enrolled 54% of patients with fibrosis ≥F3 and nine patients with CH; moreover, 54% of patients had failed under previous dual anti-HCV therapy after LT. The higher rate of SVR24 achieved with PI-triple therapy (compared to historical reports) is consistent with the results reported by others with SVR12 [12,13,14,15]. The American Crush-C group recently reported an SVR12 rate of 63% (51/81) in a cohort of difficult-to-treat patients whose characteristics were similar to those in our cohort [13]. Interestingly, in our patients we observed a marked improvement in the clinical and biological parameters of patients with high bilirubin levels and high MELD scores at baseline. Among the 45 patients with a baseline bilirubin level >17μmol/L, we observed a stabilization or improvement in 88% of them. This improvement was particularly significant in CH and F4 patients, as their median MELD scores decreased in a statistically significant manner between baseline and EOT (*P* = 0.02 and *P* = 0.04, respectively).

Predictive factors of virological clearance were also investigated in our series. To discuss the potential superiority of one of the two drugs is not appropriate here because the patients were not randomized and the choice of BOC or TVR by the investigators was mainly dependent on the accessibility of the two drugs under the French Temporary Authorization for Use system. Achieving an EVR (OR = 49.4, 95%CI [5.7–421.3], *P<*0.00001) was the strongest predictor of an SVR24. Burton *et al*. also found that achieving EVR is the best predictor of SVR12 using TVR after LT. Here in, we comfort this finding and confirm that this result could be applied to a population using BOC [13]. In the absence of a complete EVR, the chance of achieving an SVR24 was only 3%. In this context, we propose to redefine the stopping rules for triple therapy in transplant patients: if the viral load remains detectable at W12, treatment should be discontinued. Unsurprisingly, under univariate analysis, other factors known to negatively impact the response to treatment during dual therapy have been identified, including the status of the recipient with respect to IL28B, being overweight defined as a BMI>25, a null response to prior therapy and the fibrosis stage, mainly F4 patients and CH [5,6,20]. Indeed, we were able to confirm that hepatic impairment significantly decreased the chances of achieving an SVR. Both a bilirubin level <17μmol/L and an albumin level >35g/L were independent factors associated with an SVR (*p* = 0.014 and *p* = 0.037, respectively). Consequently, triple therapy should be proposed at an earlier stage of liver graft disease due to a recurrence of HCV. A starting dose of RBV ≥800mg/day was also an independent factor for an SVR24 (*p* = 0.003). These results argue in favor of introducing a full dose of RBV to ensure renal function, and using hematopoietic growth factors as first-line treatment for cytopenia in order to maintain the maximum tolerated dose of RBV [21]. If no premature discontinuation of treatment due to an SAE was necessary, this also was an independent predictor for an SVR24 (OR = 6.3, 95%CI [1.3–31.1], p = 0.02). The IS regimen impacts the treatment response rate because the use of cyclosporine and the avoidance of steroids were both associated with an SVR24 under univariate analysis (*p* = 0.01 and *p* = 0.04, respectively). One hypothesis is the potential antiviral effect of cyclosporine, as demonstrated *in vitr*o [22]. Less significant drug-drug interactions between cyclosporine and CNI, enabling easier treatment management, might be another, more relevant, hypothesis [8,9,10].

As well as offering findings on antiviral efficacy, our study has also provided comprehensive data on the tolerance of triple therapy with BOC and TVR. The poor tolerance of anti-HCV therapy may have serious consequences, such as premature discontinuation and exposure to severe or fatal events. We had already reported in an interim analysis that the most frequent adverse events were represented by anemia, thrombocytopenia and neutropenia [11]. A drop in hemoglobin levels to below 10g/dL was observed in 95% of patients, despite a reduction in the RBV dosage in 70% of the cohort, and the use of EPO in 94% of patients at an early stage (means: 7 W and 4 W, respectively). Forty percent of the patients required a transfusion (mean: 4.2 units per patient). We therefore strongly recommend introducing EPO at an early stage, even before the initiation of anti-HCV therapy if the patient experienced haematological toxicity during a previous course of antiviral therapy.

Delaying the introduction of EPO exposes the patient to more profound anemia and a risk of premature treatment discontinuation. Neutropenia (<1G/L) and thrombocytopenia (<50G/L) are less common than anemia, but were more frequently observed in the TVR group than the BOC group in our study (*P* = 0.004 and *P* = 0.02, respectively). A reduction in the PEG-IFN dose was observed in 39% of patients, and the use of growth factors was infrequent (G-CSF: n = 15 (18%), Eltrombopag: n = 5 (6%)). Although it affected 95% of patients, cytopenia was not an obstacle to the pursuit of treatment and only 3% and 13% of patients stopped treatment for this reason in the BOC and TVR groups, respectively.

A decrease in the eGFR during therapy (median decrease: 7.68 mL/min with BOC and 8.53 mL/min with TVR, *P* = ns) was observed [23]. There was no difference between the two groups, but a trend towards a higher re-hospitalization rate because of acute kidney failure in the TVR group than in the BOC group (6 *versus* 1; *P* = 0.23). The duration of impaired renal function did not exceed the duration of treatment, and the eGFR rose when the PI was withdrawn, and normalized after the discontinuation of PEG-IFN/RBV.

The occurrence of episodes of infection affected a third of patients in our cohort. This major problem was the cause of death in four patients (5%). Not surprisingly, CH patients were six times more exposed to infectious episodes (*p* = 0.05). Prophylactic antibiotics should be investigated in this subgroup of patients before the initiation of anti-HCV therapy. A fall in the platelet count to <50G/L during therapy was an independent factor for both infectious episodes and SAE (*p* = 0.02 and *p* = 0.01, respectively). Thrombocytopenia has recently been shown to be associated with a poorer tolerance of IFN-based anti-HCV therapy in severe, non-transplant patients [24]. The use of Eltrombopag has been shown to increase the platelet count in thrombocytopenic patients with HCV and advanced diseases, leading to significantly higher SVR rates [25]. Taken together, these data argue for the use of platelet growth factors during therapy, and most importantly a significant drop in the platelet count should alert physicians to an increased risk of SAE or infections. A strict monitoring of portal flow should be ensured during the treatment, especially in patients with portal hypertension.

Despite the feasibility of the using such drugs, and the improvement in efficacy achieved when compared to an historical cohort in the liver transplant setting, safety profile of these regimens limits their application given the rapidly evolving field of HCV therapy. Several second generation DAA have now been approved, and preliminary reports in non-transplant patients have shown their remarkable efficacy and, most importantly, their good safety profile [26]. In a prospective, multicenter, open-label pilot study enrolling 40 liver transplant patients, a combination of sofosbuvir and RBV was able to achieve an SVR12 in 70% of patients. The most common adverse events were fatigue (30%), diarrhea (28%), and headache (25%). In addition, 20% of the subjects experienced anemia [27]. The real highlight of these second generation DAA is that their combination enables treatment without the need to use PEG-IFN, and possibly RBV [28,29]. However, data in the liver transplant setting remain limited.

Although our study offers some key messages regarding the use of first generation PI in liver transplant patients, it is likely that in the near future this strategy will be outdated western countries. Three important points need to be stressed regarding future practice. Firstly, some countries have access to these treatments and still use them. This study is the only one to have provided predictive factors for SVR24 in transplanted patients treated with triple therapy. Our data argue in favor of initiating treatment during the early stages of HCV recurrence with a full dose of PEG-IFN/RBV. The risk-benefit balance advocates for discontinuing treatment when an EVR is not achieved. Secondly, although a satisfactory safety profile is likely using IFN-free regimens, RBV continues to be used, mainly in difficult-to-treat patients. Our findings argue in favor of initiating early hematological growth factor therapy in order to avoid dose reduction or treatment discontinuation. Finally, some DAA (simeprevir, daclatasvir ombitasvir, dasabuvir, asunaprevir) are also metabolized by CYP3A4. First-generation triple therapies have raised awareness to drug-drug interactions in the setting of HCV therapy. Although weaker with second-generation treatments, they remain an issue with some regimens, especially when second-generation protease inhibitors are used [30,31]. In patients with a severe recurrence, the cautious use of certain DAA is warranted in the event of hepatic impairment [32].

To conclude, triple therapies are now being widely used, given their performance in terms of efficacy when compared to previous dual therapy. Nevertheless, their safety profile, specifically in difficult-to-treat patients, remains poor. In light of these findings, we believe that caution is of the essence regarding the approval of second generation DAA.

## Appendix: Members of the AFEF prospective group of liver transplantation

Laurent Alric, Teresa-Maria Antonini, Rodolphe Anty, Camille Besch, Danielle Botta-Fridlund, Yvon Calmus, Paul Carrier, Audrey Coilly, Filomena Conti, Marilyne Debette-Gratien, Thomas Decaens, Sebastien Dharancy, Jean-Charles Duclos-Vallée, Jérôme Dumortier, François Durand, Christophe Duvoux, Stéphanie Faure, Cyrille Feray, Claire Francoz, Olivier Guillaud, Pauline Houssel-Debry, Géraldine Lamblin, Marianne Latournerie, Pascal Lebray, Vincent Leroy, Hélène Montialoux, Georges-Philippe Pageaux, Sylvie Radenne, Bruno Roche, Didier Samuel, Moana Gelu-Simeon, Rodolphe Sobesky.

## Supporting Information

S1 Table Description of the 25 episodes of infections. (DOCX)

## Abbreviations

AFEF: French Association for the Study of the Liver
ALT: alanine aminotransferase
bid: twice daily (*bis in die*)
BOC: boceprevir
BPAR: biopsy proven acute rejection
cEVR: complete early virological response
C0: Trough blood concentration
CH: cholestatic hepatitis
CNI: calcineurin inhibitors
DAA: direct acting agents
eGFR: estimated glomerular filtration rate
EOT: end of treatment response rate
EPO: erythropoietin
eRVR: extended rapid virological response
EVR: early virological response
F: Female
G1: genotype 1
GGT: gamma-glutamyl transferase
HBV: hepatitis B virus
HCC: hepatocellular carcinoma
HCV: hepatitis C virus
HIV: human immunodeficiency virus
IL: interleukin
INR: International Normalized Ratio
IS: immunosuppressive drugs
Kg: Kilogram
LT: liver transplantation
M: Male
MELD: Model for End-stage Liver Disease
MMF: mycophenolate mofetil
NA: not available
NR: non-response
PCR: polymerase chain reaction
PEG-IFN: pegylated interferon
PI: protease inhibitors
qd: once a day (*quaque die*)
RBV: ribavirin
RVR: rapid virological response
SAE: serious adverse event
SVR12: sustained virological response 12 W after the end of therapy
SVR24: sustained virological response 24 W after the end of therapy
tid: three times a day (*ter in die*)
TVR: telaprevir
VB: virological breakthrough
VL: viral load
VR: virological response
