# Supplementary material for: Multicenter Experience with Boceprevir or Telaprevir to Treat Hepatitis C Recurrence after Liver Transplantation: When Present Becomes Past, What Lessons for Future?
Source: PLoS One. 2015 Sep 22;10(9):e0138091. doi: 10.1371/journal.pone.0138091 (PMC4578772; doi:10.1371/journal.pone.0138091)
Supplement: S1 Table — (DOCX) [file pone.0138091.s001.docx]

**S1 Table: Description of the 25 episodes of infections**

| **Infection site** | **Delay from baseline (Week)** | **Protease inhibitor** | **Underlying cirrhosis or cholestatic hepatitis** | **Treatment discontinuation due to this infection** | **Outcome** |
| --- | --- | --- | --- | --- | --- |
| Lung | 1 | Telaprevir |  | No |  |
|  | 2 | Telaprevir | Cirrhosis | Yes |  |
|  | 3 | Telaprevir |  | Yes |  |
|  | 4 | Telaprevir |  | No |  |
|  | 8 | Telaprevir |  | Yes | Death |
|  | 8 | Telaprevir | Cholestatic hepatitis | Yes |  |
|  | 1 | Boceprevir | Cirrhosis | No |  |
|  | 24 | Boceprevir |  | No |  |
|  | 25 | Telaprevir |  | No |  |
|  | 3* | Telaprevir | Cholestatic hepatitis | Yes |  |
|  | 2* | Boceprevir | Cirrhosis | Yes | Death |
|  | 24* | Boceprevir | Cirrhosis | Yes |  |
| Peritonitis | 24 | Boceprevir |  | Yes | Death |
|  | 24 | Boceprevir |  | Yes |  |
| Pylephlebitis | 4 | Telaprevir |  | Yes |  |
| Sinus | 11 | Telaprevir | Cholestatic hepatitis | Yes |  |
| Skin | 6 | Telaprevir | Cirrhosis | Yes |  |
|  | 16 | Boceprevir |  | No |  |
|  | 16 | Boceprevir |  | No |  |
| Urinary tract | 2 | Telaprevir | Cholestatic hepatitis | Yes | Death |
|  | 4 | Boceprevir |  | No |  |
|  | 12 | Boceprevir |  | No |  |
|  | 12 | Boceprevir |  | No |  |
|  | 12 | Boceprevir | Cholestatic hepatitis | No |  |
|  | 42 | Boceprevir |  | No |  |

*Opportunistic infections: 1 Cytomegalovirus infection, 1 aspergillosis and 1 pneumocystis pneumonias
